# Enhanced γ-Glutamyltranspeptidase Imaging That Unravels the Glioma Recurrence in Post-radio/Chemotherapy Mixtures for Precise Pathology via Enzyme-Triggered Fluorescent Probe

**DOI:** 10.3389/fnins.2019.00557

**Published:** 2019-05-31

**Authors:** Ben Shi, Zhenyu Zhang, Chuanjin Lan, Bao Wang, Shangchen Xu, Mingxu Ge, Ge Xu, Tianli Zhu, Yingchao Liu, Chunchang Zhao

**Affiliations:** ^1^Key Laboratory for Advanced Materials and Institute of Fine Chemicals, School of Chemistry and Molecular Engineering, East China University of Science and Technology, Shanghai, China; ^2^School of Medicine, Shandong University, Jinan, China; ^3^Department of Neurosurgery, Shandong Provincial Hospital Affiliated to Shandong University, Jinan, China

**Keywords:** biomarker, γ-glutamyltranspeptidase, fluorescent probe, glioma recurrence, pathological diagnosis

## Abstract

Accurate pathological diagnosis of gliomas recurrence is crucial for the optimal management and prognosis prediction. The study here unravels that our newly developed γ-glutamyltranspeptidase (GGT) fluorescence probe (Figure 1A) imaging in twenty recurrent glioma tissues selectively recognizes the most malignant portion from treatment responsive tissues induced by radio/chemo-therapy (Figure 1B). The overexpression of GGT in recurrent gliomas and low level in radiation necrosis were validated by western blot analysis and immunohistochemistry. Furthermore, the ki-67 index evaluation demonstrated the significant increase of malignancy, aided by the GGT-responsive fluorescent probe to screen out the right specimen through fast enhanced imaging of enzyme activity. Importantly, our GGT-targeting probe can be used for accurate determination of pathologic evaluation of tumor malignancy, and eventually for guiding the following management in patients with recurrent gliomas.

## Introduction

Glioma, one of the most common types of brain tumors, are characterized by a high fatality rate and poor prognosis (Ceyssens et al., 2006; Corti et al., 2010). The standard clinical care for newly diagnosed malignant gliomas involves surgical intervention, postoperative adjuvant radiation therapy and chemotherapy. Notably, even after such a comprehensive treatment, tumors invariably progress to high-grade glioma tumors after radio/chemotherapy (DeAngelis, 2001; Eyupoglu et al., 2013; Gaudin et al., 2016; Fang et al., 2017; Gao et al., 2017). Postoperative adjuvant radiation therapy and chemotherapy can lead to the incidence of radiation necrosis and gliosis (Han et al., 2007). It is difficult to distinguish post-radiation responsive tissues from tumor recurrence in clinical practice. Tissue samples obtained by biopsy or resection are randomly collected according to the operator’s experience for histopathology evaluation, leading to misdiagnosis because of the selective bias (Hanigan and Ricketts, 1993; Han et al., 2007). Furthermore, previous studies have demonstrated the importance of histopathology as an independent prognostic factor, (He et al., 2016) and incorrect pathologic diagnosis leads to poor management of glioma patients.

**FIGURE 1:**
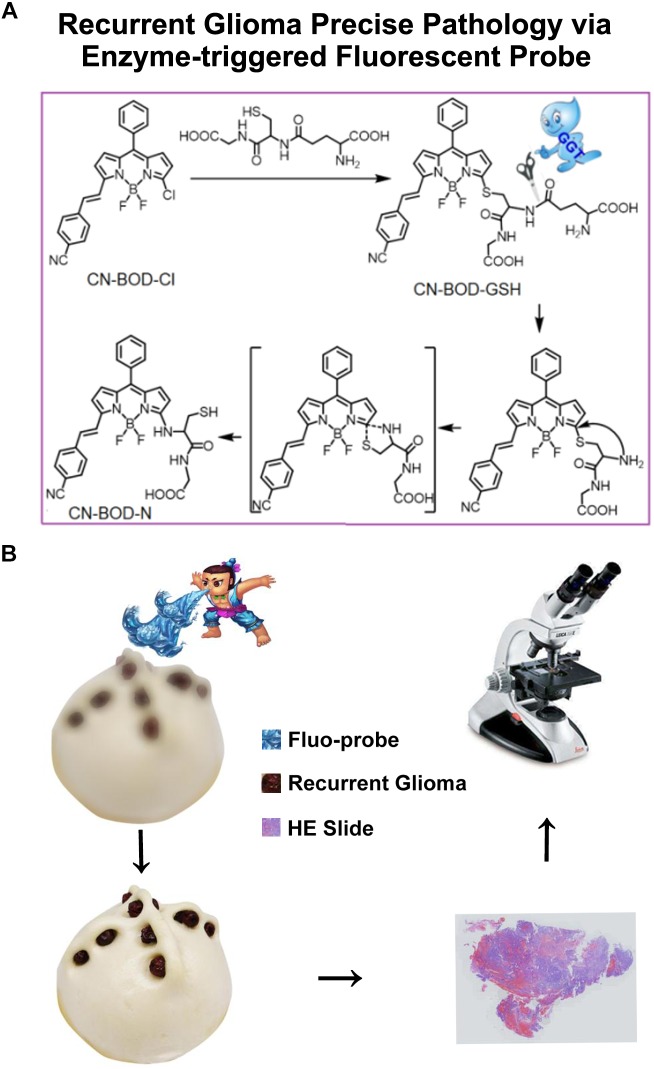
**(A)** Schematic illustrations of GGT-catalyzed transformation of CN-BOD-GSH to CN-BOD-N and **(B)** the cartoon graph showed the process of the visualization of recurrent glioma for precise pathology diagnosis.

Standard magnetic resonance imaging (MRI) with gadolinium-based contrast agents cannot reliably differentiate tumor recurrence and post-radiation necrosis, as the signals of treatment-related effects on MRI resemble those of tumor recurrence (Han et al., 2007; Huse and Holland, 2010; Hou et al., 2014). Several complicated imaging modalities, combining morphologic, metabolic, and functional MR imaging parameters, demonstrated increased diagnostic value (Johnson et al., 2014). However, the reliabilities of these techniques are difficult to confirm by corresponding pathology evaluation (Jung et al., 2013). Additionally, these advanced MRI modalities are subject to only concerning the following up and pre-operative evaluation but are helpless for the pathology diagnosis, which depends on the right portion of recurrence (Kim et al., 2014).The cell surface enzyme γ-glutamyltranspeptidase (GGT) selectively catalyzes the metabolism of glutathione (GSH) to supply cysteine, which is highly demanded by malignant tumor cells, thus associating with tumorigenesis (Liu et al., 2008; Li et al., 2015). Indeed, overexpression and elevated activity of GGT has been detected in various cancers, such as cervical cancer, ovarian cancer and glioma (Mullins et al., 2005; Liu et al., 2018). Relatively low expression of GGT is observed in healthy tissues compared with cancer tissues, making GGT a suitable biomarker for cancer tissue detection (Pompella et al., 2006; Petrik et al., 2008; Niu et al., 2012; Sounni and Noel, 2013; Niu et al., 2015; Nishihara et al., 2016; Soliman and Yussif, 2016; Tong et al., 2016). As post-radiation change lesions are composed of gliosis and glial scar that are asymptomatic, we speculate that these lesions would exhibit low GGT activity compared with recurrent gliomas but it is minimally expressed in radiation necrosis. Furthermore, we demonstrate that GGT can serve as a potential biomarker for the differentiation of tumor recurrence and post-radiation lesions using a smart molecular imaging probe through direct monitoring of GGT activity. Importantly, we prove the potency of GGT activity mapping for identifying accurate tissue samples for precise pathologic evaluation and eventually for prognosis prediction.

## Materials and Methods

### Patient Cohort

A prospective non-randomized study design was used. From March 2015 to October 2017, patients with newly appeared or enlarged contrast-enhancing lesions, during the follow-up after radio/chemo-therapy, underwent the second operation. All patients provided written informed consent. This study was approved by the Shandong University Institutional Review Board (NO.2015-051). A total of 20 patients (7 males, 13 females; mean age: 45.5 years; age range: 13–65 years) were enrolled with the pathology diagnosis after resection. Among the 20 patients, 12 patients were treated with surgery, chemotherapy and radiotherapy, and 8 patients were treated with surgery and radiotherapy. Tumor samples were diagnosed by neuropathologists who were blinded to patient data, and classification was performed using the World Health Organization (WHO) system. Clinical data, including gender, age, follow-up, and outcome, were obtained from the medical records. The clinical characteristics of the patients are shown in Supplementary Table S1.

### Cell Lines

The human glioblastoma tumor cell lines U87 and U251 were obtained from the Cell Bank of the Type Culture Collection of the Chinese Academy of Sciences (CBTCCCAS; Shanghai, China). Cells were cultured in Dulbecco’s modified essential medium (DMEM) supplemented with 10% fetal bovine serum (FBS, Gibco, United States), penicillin (100 units/mL, Gibco) and streptomycin (100 g/mL, Gibco) at 37°C in an incubator flushed continuously with 5% CO_2_. Cells were seeded at a density of 2.5 × 10^5^ cells in 75 cm^2^ flasks. Temozolomide (TMZ, Sigma-Aldrich) in 10% dimethyl sulfoxide (DMSO, Sigma-Aldrich) was added (20 μM) and cells were cultured for 48 h. Two hours after TMZ treatment, cells were irradiated with 2 Gy on an X-ray generator (120 kV, 22.7 mA, variable time; GE Inspection Technologies, Hürth, Germany), representing a daily dose in glioblastoma multiforme therapy. Irradiation was repeated on 3 consecutive days to achieve a clinically relevant total dose of 6 Gy. Colonies containing more than 50 cells were counted as a representation of clonogenic cells and were plated in triplicate in 60-mm dishes (Nunc Thermo Fisher Scientific Inc., Waltham, MA, United States). The radio/chemo-resistant cell lines (U87R and U251R) were cultured in DMEM supplemented with 10% FBS in a humidified cell incubator with 5% CO_2_ at 37°C for about 2 weeks. Exponentially growing cells were used for experiments.

Normal human astrocyte (NHA) cells plated at 7500 cells/cm^2^ were incubated at 37°C, 5% CO_2_, 95% O_2_ and 95% humidity. After 4–6 days, astrocyte cells were harvested.

Cells (U87R, U251R, and NHAs) were lysed in NP40 lysis buffer (50mM Tris, pH 7.4, 250 mM NaCl, 5 mM EDTA, 50 mM NaF, 1 mM Na_3_VO_4_, 1% Nonidet P40, 0.02% NaN_3_) containing protease inhibitor cocktail tablets and phenylmethanesulfonyl fluoride (PMSF). Tumor tissues were cleaned with normal saline and then lysed using lysis buffer.

### Confocal Fluorescence Imaging

NHA, U251R, and U87 cells were loaded with the fluorescent probe CN-BOD-GSH (10 μM) for 30 min. Fluorescence images were performed using the A1R^+^/A1^+^ confocal laser microscope system and the new resonant scanner supporting both high speed and high definition imaging. The excitation wavelength was 514 nm and the emission was collected between 580 and 650 nm. For the inhibitory effect assay, cells were pre-treated with GGsTOP (100 μM) for 30 min and then incubated with CN-BOD-GSH (10 μM) for 30 min.

### GGT Probe-Assisted Precise Pathology Diagnosis

Human recurrent glioma specimens were collected from the 20 patients who underwent neuronavigation-guided tumor resection by GGT activity mapping. The selected samples were analyzed *ex vivo* with an IVIS *in vivo* imaging system. The excitation wavelength was 500 nm and the emission was 580–650 nm. All fluorescence data were collected and analyzed by the fluorescence intensity analysis software coupled with the IVIS system. Healthy brain tissues from the ventriculostomy for ventricular meningioma resection were used to determine background fluorescence.

The collected glioma specimens were first sprayed with 150 μL 1 mM/L of CN-BOD-GSH (10 mM/L for stock solution) onto the surface of the specimens, and snapshot images were captured 5 min later. We then subtracted the fluorescent signals in specimens obtained in normal brain tissues as the background. Tissues with levels of fluorescence similar to those of background were assigned as post-irradiation lesions. In contrast, recurrent tumor tissues showed brighter fluorescent signals, and we continued to attenuate the bright fluorescence of the experimental samples one by one until only residual fluorescence foci (the highest fluorescence portion) remained. Biopsy samples containing these fluorescent foci were chosen for pathology evaluation.

### Histological and Immunohistochemistry Evaluation

Samples for histopathological assessment were selected according to the neurosurgeon’s experience. Samples containing fluorescent foci and post-irradiation lesions, as described above, were chosen for histological and immunohistochemistry analysis in parallel. These biopsy samples were immediately placed in zinc-formalin for 4–6 h, dehydrated in a series of graded alcohols and then embedded with low-temperature paraffin for histological analysis. Specimens containing fluorescent foci and the post-irradiation lesions were fixed for at least 24 h in 10% neutral buffered formaldehyde. Paraffin-embedded 4 μM sections were stained with hematoxylin and eosin and routine immunohistochemistry for determination of GGT expression and Ki-67 index. Immunohistochemical investigation was performed with antibodies anti-human GGT Mab (1:400, clone DO-7, Abcam, Cambridge, United Kingdom) and Ki-67 Mab (mouse anti-human, 1:100, clone MIB-1), followed by peroxidase-DAB terminal staining (EnVision+Dual Link System-HRP). Two neuropathologists blinded to the clinical categorization of these samples independently evaluated the results. In case of any disagreement, they discussed the data and drew a consensus for final diagnosis. Tissues showing features of the glioma diagnosis criteria were assigned as recurrent tumors while those exhibiting features of necrosis or gliosis were defined as radionecrosis or gliosis.

### GGT Fluorescence Probe Chemical Synthesis

#### Synthesis of compound CN-BOD-Cl

To a solution of compound 4 (410 mg, 2 mmol) in 10 mL ClCH_2_CH_2_Cl, we added POCl_3_ (4 ml) at 0°C. The resulting mixture was stirred for 0.5 h at 0°C and 12 h at room temperature. Compound 3 (194 mg, 1 mmol) in 10 mL ClCH_2_CH_2_Cl was then added, and the resulting solution was refluxed for 0.5 h and then cooled to room temperature. Saturated NaHCO_3_ solution was then added at 0°C. The ClCH_2_CH_2_Cl phase was dried over Na_2_SO_4_ and evaporated under vacuum to obtain the crude product, compound 5, which was used for further reaction without purification. Compound 5 was dissolved in 30 mL anhydrous CH_2_Cl_2_ and then 0.5 mL Et_3_N and 1 mL boron fluoride ethyl ether were added. The mixture was stirred for 5 h at room temperature. The reaction mixture was then washed with H_2_O three times and the organic phase was dried over Na_2_SO_4_. After removal of CH_2_Cl_2_, the resulting residue was purified by column chromatography on silica gel to afford the target compound CN-BOD-Cl (35 mg, 8%). ^1^HNMR (400MHz, CDCl_3_), δ = 7.85–7.81 (d, 1H), 7.72–7.66 (q, 4H), 7.59–7.57 (m, 1H), 7.55–7.50 (m, 4H), 7.36–7.32 (d, 1H), 6.99–6.98 (d, 1H), 6.93–6.92 (d, 1H), 6.81–6.80 (d, 1H), 6.43–6.42 (d, 1H). HRMS (ESI, m/z), calculated for C_24_H_16_BClF_2_N_3_ [M + H]^+^: 430.1094, Found: 430.1089.

#### Synthesis of compound CN-BOD-GSH

To a solution of compound CN-BOD-Cl (20 mg) in 150 mL CH_3_CN-PBS (CH_3_CN:PBS = 1:1, pH = 7.40), GSH (130 mg) was added, and the resulting mixture was stirred for 12 h at 41°C. After removal of CH_3_CN, crude product was obtained by centrifugation. The crude product was washed with H_2_O and CH_2_Cl_2_ three times, followed by dissolving in MeOH. The solid was filtrated to afford a clear solution that was evaporated under vacuum to give the desired compound CN-BOD-GSH (21 mg, 64%) in Supplementary Figure S1. ^1^HNMR (400MHz, CD_3_OD), δ = 7.83–7.79 (m, 1H), 7.78–7.73 (m, 4H), 7.61–7.59 (m, 1H), 7.58–7.55 (m, 4H), 7.48–7.44 (d, 1H), 7.09–7.08 (d, 1H), 6.95–6.94 (d, 1H), 6.91–6.90 (d, 1H), 6.80–6.79 (d, 1H), 4.80–4.77 (dd, 1H), 3.85–3.78 (m, 2H), 3.77–3.74 (m, 1H), 3.63–3.59 (t, 1H), 3.45–3.39 (dd, 1H), 2.64–2.51 (m, 2H), 2.16–2.11 (dd, 2H). ^13^C NMR 174.52, 174.22, 172.53, 169.80, 156.14, 141.30, 136.52, 133.64, 132.96, 132.50, 132.41, 130.20, 129.93, 128.52, 128.32, 128.21, 127.77, 127.34, 122.09, 118.35, 116.40, 111.11, 110.45, 72.12, 60.85, 29.35, 26.35. HRMS (ESI, m/z), calcd for C_34_H_30_BF_2_N_6_O_6_ [M-H]^-^: 699.2009, Found: 699.2017.

### Statistical Analysis

All data were expressed as mean ± SD. Analysis of variance (ANOVA) was used to test the difference between three or more groups. The Ki-67 index and the fluorescence intensities of fluorescent foci (identified using CN-BOD-GSH) were compared using Spearman correlation analysis. Tumor grades obtained from different methods were compared by paired *t*-test. Differences were considered statistically significant at a *P*-value of less than 0.05.

## Results

### The GGT Expression in Post Radio/Chemo-Therapy Related Tissues and Cell Lines

**FIGURE 2:**
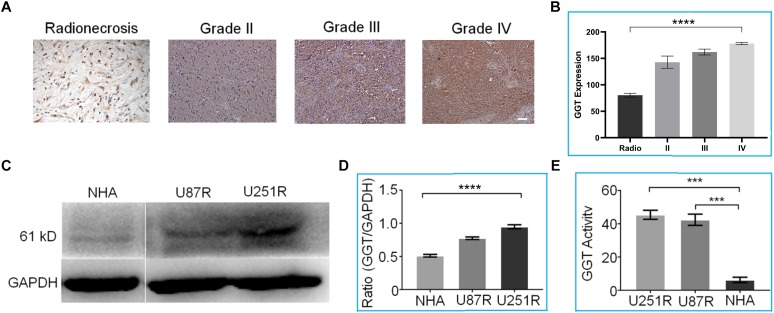
**(A)** Representative immunohistochemical staining of GGT in various recurrent gliomas and post-radio/chemotherapy lesions. Scale bar = 20 μm. **(B)** Average GGT expression levels in recurrent gliomas and post-irradiation lesions. **(C)** Representative western blot of GGT expression in U87R, U251R and NHA cell lines. GAPDH was used as the endogenous control. **(D)** Quantification of relative GGT expression from **(C)** in the indicated cell lines. **(E)** GGT activity was evaluated in the indicated cell lines (U87R, U251, and NHA) using a Beckman Coulter AU5800 Clinical Chemistry Analyzer. IHC Scares bars (20×) 10 μM. Error bars represent the mean ± SD. *P*-values are two side ANOVA. ^∗∗∗^*P* < 0.001,^∗∗∗∗^*P* < 0.01 in all experimental groups.

First, we sought to investigate the expression levels of GGT in recurrent gliomas and post-radio/chemotherapy lesions. Immunohistochemical analysis of tissues taken from 20 recurrent gliomas was performed to detect GGT expression. As shown in Figure 2A, up-regulation of GGT was observed in recurrent gliomas irrespective of their pathologic grades (WHO II, III and IV). In contrast, the expression of GGT remained low in the post-radio/chemotherapy necrosis and gliosis. Obviously, there exists a significant correlation between GGT expression and pathologic grade of the recurrent tumors (Figure 2B). Similar to recurrent tumors, radio/chemo resistant glioma cell lines U87R and U251R exhibited overexpression of GGT by western-blot (Figure 2C,D) and elevated activity, compared with NHAs as the normal cell control model (Figure 2E).

### The Character of GGT Reactive CN-BOD-GSH Probe

To accurately identify regions of recurrent lesions for precise pathology evaluation, we developed a new fluorescent *in situ* targeting probe (CN-BOD-GSH) that specifically monitors GGT activity, enabling the differentiation of tumor recurrence and post-radio/chemotherapy lesions based on the difference in GGT expression levels and activities. The GGT-responsive fluorescent probe was composed of GSH as a GGT-specific substrate and a boron-dipyrromethene (BODIPY) platform as the fluorescent reporter.

**FIGURE 3:**
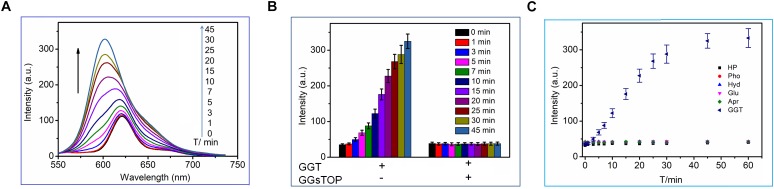
**(A)** Time-dependent fluorescence changes of CN-BOD-GSH (5 μM, HEPES + 10% DMSO) in the presence of GGT (30 mU/mL). **(B)** The fluorescence intensity changes at various time points with CN-BOD-GSH (600 nm) upon addition of GGT alone or with GGsTOP. **(C)** Time-dependent fluorescence intensity at 600 nm of CN-BOD-GSH toward various biological analytes.

We first tested the fluorescence response of CN-BOD-GSH toward GGT in buffer solutions. As shown in Figure 3A, GGT introduced an obvious hypsochromic shift of the emission maximum from 620 to 600 nm, accompanied by a dramatic fluorescence enhancement. The fluorescence change reached completion within 45 min. However, the fluorescence changes were greatly attenuated by pre-treatment of GGT with GGsTOP (Figure 3B), a highly specific GGT inhibitor, (Urano et al., 2011) demonstrating the vital role of GGT in activating the fluorescent signal variations. The GGT-triggered fluorescence change was attributed to the catalyzed cleavage of the γ-glutamyl bond in GSH to release a free amino group (Supplementary Figure S2), which spontaneously promotes the formation of amino-substituted BODIPY via a five-membered cyclic transition state (Wang et al., 2015a, 2018). In buffer solution at pH 7.4, the catalytic ability of GGT toward CN-BOD-GSH showed a Michaelis constant (K_m_) of 9.78 μM and V_max_ of 2.75 μM min^-1^ (Supplementary Figure S3). Note that the evident fluorescence enhancement was selectively produced only by GGT, while other biological analyses, including collagen hydrolase, phosphatase, aprotinin, glucoamylase and human serum albumin, gave minimal fluorescence changes (Figure 3C). The high selectivity of the probe for GGT ensures accurate enzyme assay results in translational medicine applications.

### Therapy Resistant Glioma Cell Line Fluorescence Imaging by Confocal Fluorescence Microscopy

**FIGURE 4:**
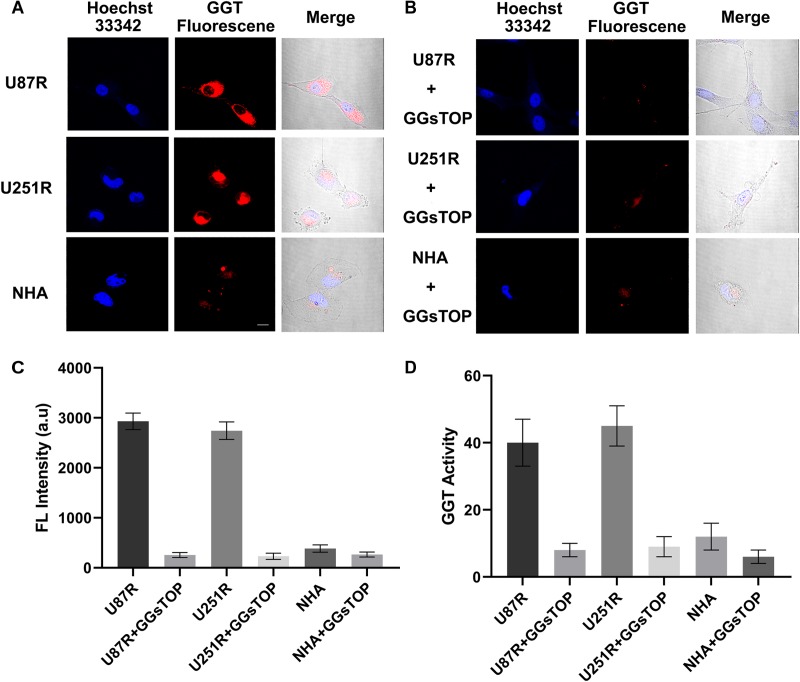
**(A)** Confocal fluorescence imaging of indicated cell lines after incubation with CN-BOD-GSH (10 μM) for 30 min. U87R, U251R and NHA cells pretreated with a GGT inhibitor (GGsTOP 100 μM) for 30 min and then loaded with NC-B-Cys-Gly (10 μM) for 30 min. **(C)** Average fluorescence image intensities from **(A,B)**. **(D)** GGT activity in the cellular lysates of U251R and U87R glioma cells and NHA cells in the absence and presence of GGsTOP. The fluorescence signals are the mean ± SD of three independent measurements. Statistical analysis was performed using the Student’s *t*-test, ^∗∗∗^*P* < 0.001. All the images share the same scale bar of 20 μm.

The cell lines (U87R, U251R and NHA) were then incubated with CN-BOD-GSH and examined by confocal fluorescence microscopy. U87R and U251R glioma cell lines exhibited bright red fluorescence images after incubation with the probe for 30 min while in contrast, HHAs cells that express low levels of GGT exhibited weak red fluorescence (Figure 4A). The red fluorescence was obviously attenuated by the addition of GGT inhibitor GGsTOP (Figure 4B), which resulted in the reduced fluorescence intensity in all experimental cell lines (Figure 4C). These results were also validated by the test of GGT activity in U87R, U251R glioma cells and NHAs, before and after the incubation of GGsTOP (Figure 4D). Together these data prove the reliability of CN-BOD-GSH for differentiation of glioma cell lines and normal glia cells using GGT as a biomarker. The cytotoxicity of the probe was also exampled toward NHA cells using CCK-8 (Cell Counting Kit) assays. CN-BOD-GSH treatment for 24 h did not significantly decrease cell viability even at a final concentration of 100 μM (Supplementary Figure S4), suggesting its low biological cytotoxicity.

### Fast Detection of Tissues for Precise Pathology by Enhanced GGT Imaging

**FIGURE 5:**
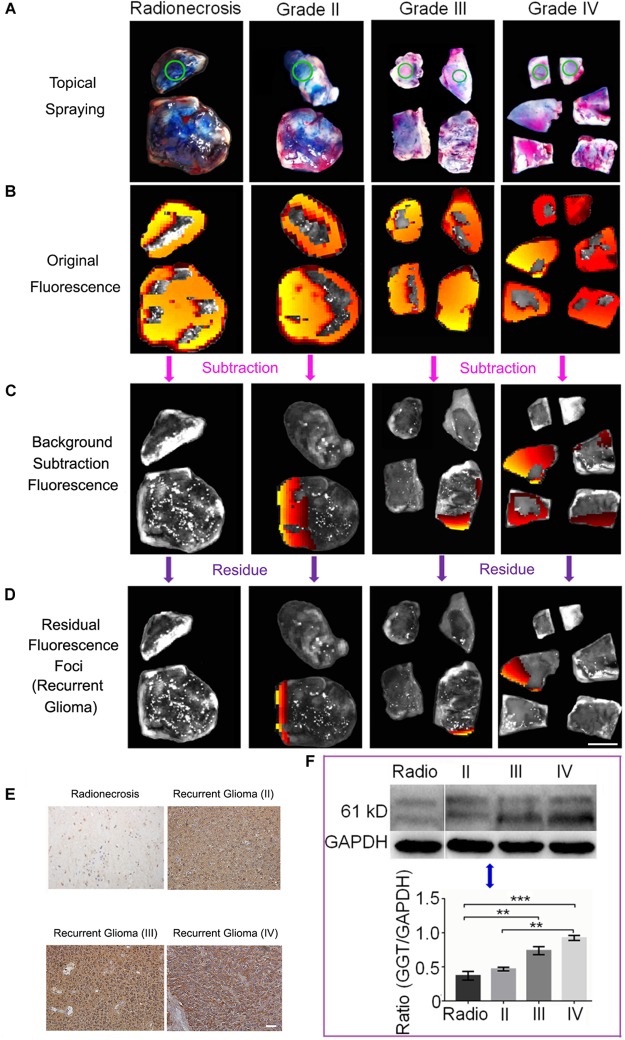
**(A)** Topical spraying of the probe on specimens for the imaging experiments. Green circles represent the healthy tissue. **(B)** Initial fluorescence images recorded after applying CN-BOD-GSH (150 μL, 1 mM in DMSO). **(C)** The subtraction fluorescence in various tissues from the corresponding control tissue to sort out the non-tumorous radionecrosis or gliosis. **(D)** Continual fluorescence subtraction eventually yielded the fluorescent foci to identify the most malignant portion used for pathological diagnosis. **(E)** Immunohistochemical staining of GGT expression in selected by fluorescence foci as recurrent gliomas and radionecrosis. **(F)** Western blot analysis of GGT expression in Grade II, III, and IV recurrent gliomas and radionecrosis. The western-blot signals are the mean ± SD of three independent measurements. Statistical analysis was performed using the two side ANOVA, ^∗∗^*P* < 0.01,^∗∗∗^*P* < 0.001. All the IHC images and tissue samples share the same scale bar of 20 μm and 10 mm, respectively.

We then further investigated the power of GGT-targeted CN-BOD-GSH for screening accurate tissue for pathology diagnosis. Clinical tissue specimens from 20 patients who underwent the second glioma resection were used to examine the ability of the probe to accurately identify recurrent gliomas (Supplementary Figure S5). Figure 5 shows representative imaging examples of three patients with Grade II, III, or IV recurrent tumors and one patient with post-irradiation change. A topical spraying protocol was used to apply the probe to the surface of specimens (Figure 5A,B). We used the fluorescence of healthy tissues obtained from a ventriculostomy patient as the background fluorescence and subtracted the fluorescent signals until it disappeared as the background fluorescence. The tissues with background levels of fluorescence were assigned to post-radio/chemotherapy lesions. After continuation with these subtraction procedures, we could finally identify the subtraction fluorescent signals in the tissue samples (Figure 5C) irrespective of the grades (WHO glioma grades II, III, or IV), and this will overcome the difficulty of selecting the right portion for the pathology diagnosis only with naked eyes. We continued to attenuate the relative weak and mild fluorescence regions one by one until only the last fluorescence foci remained, indicating regions with the highest GGT activity (Figure 5D). Together these results showed that the cancerous region was clearly detectable by *in situ* tracking of GGT activity in specimens containing both recurrent gliomas and post-treatment lesions.

We finally used the probe to identify and isolate biopsies from 17 samples with strong fluorescent foci and 3 patients whose samples showed background levels of fluorescence. The biopsies underwent pathology evaluations. As shown in Supplementary Table S1, all the 17 samples from fluorescent foci were diagnosed as recurrent gliomas. Among them, three patients (T08, T12, and T17) were determined to have highest grade of malignant recurrent glioblastoma multiforme (WHO IV) under the guidance of the CN-BOD-GSH probe. In comparison, T08 and T12 patients were initially diagnosed with anaplastic astrocytomas (AA, WHO III), while patient T17 was diagnosed with astrocytoma (AC, WHO II), because of the subjective sample selection bias. Note that patient T10 was diagnosed as post-radio/chemotherapy change at the beginning and then later diagnosed as anaplastic oligodentroastrocytoma (AOA, WHO III) with our new fluorescence probe method. The speculation that the high expression GGT in glioma recurrence and low expression in gliosis and radionecrosis were further verified by IHC (Figure 5E) and western blot analysis (Figure 5F). A high level of GGT expression was observed in the recurrent tumor region, while weak or faint staining was detected in the pos-treatment change. Together, these results indicated that the GGT fluorescence probe could provide more assistance for precise pathology diagnosis compared with assessment from subjective judgment.

### Enhanced Fluorescence Foci Correlate With Higher Malignancy

**FIGURE 6:**
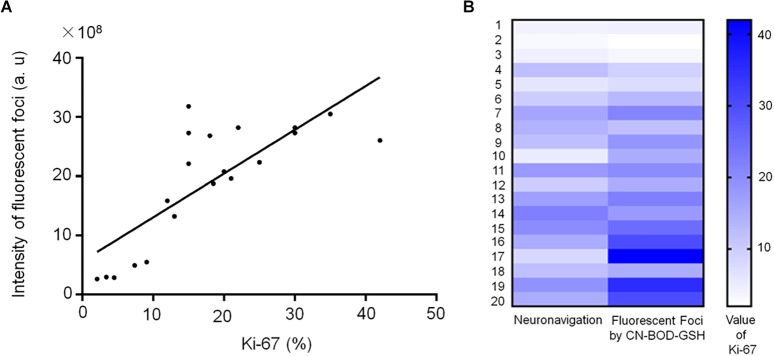
**(A)** Spearman correlation analysis indicates a significant correlation (*r* = 0.76) between Ki-67 level and the fluorescence intensities of fluorescent foci (identified using CN-BOD-GSH) in the 20 specimens from Supplementary Table S1. **(B)** Ki-67 index values obtained from fluorescent foci (identified by CN-BOD-GSH) versus those subjectively selected by neurosurgeons were analyzed with paired *t*-test.

Interestingly, Spearman’s rank correlation analysis indicated a significant correlation (*r* = 0.76) between Ki-67 index, a cellular marker for proliferation, (Wang et al., 2015b)and the foci fluorescence intensities identified by CN-BOD-GSH in the 17 specimens (Figure 6A). Using paired *t*-test, 82% of samples (14 of 17 patients with a diagnosis of recurrent tumors) showed a higher Ki-67 level than those from random and subjective selection (Figure 6B), suggesting the potential for clinic use. We further examined the clinical significance of GGT activity as an alternative prognostic marker for overall survival as noted in Supplementary Table S1. Five patients with WHO II and radionecrosis showed low GGT activity and a lower than 10% Ki-67 level, and four of these patients (4/5, 80%) exhibited better overall survival for at least 10 months. Similarly, in the WHO IV group, 91% of cases (10 of 11 patients) showed higher fluorescence intensity and Ki-67 higher than 15%, with an overall survival of less than 8 months. Together, these findings demonstrate the potency of GGT activity in predicting the proliferative nature of tumors and overall survival.

## Discussion

Accurate pathological diagnosis of recurrent tumors is crucial for the optimal management decision and therapy strategy. The highly aggressive characteristic of recurrent glioma promotes the development of powerful tools for fast and pathological diagnosis of tumor recurrence. Although MRI imaging is relatively reliable for differentiation of recurrence and post-radio/chemotherapy change, it is currently difficult or even impossible to identify the accurate area of recurrence from the resected tissue. In addition, the subjective selection of the sample for pathology by the operator also leads to an tendency to underestimate the malignancy (Jung et al., 2013; Johnson et al., 2014). This strait inspired us to develop a simple, fast and more accurate method to differentiate glioma recurrence and post-radio/chemotherapy lesions.

In this study, we observed a significant correlation between GGT activity and the pathologic grade of the recurrent tumors using western blot and immunohistochemistry. In comparison, the expression of GGT was low in the radionecrosis and gliosis. These results implied that GGT may be used as a biomarker for distinguishing tumor recurrence from radionecrosis and gliosis.

Although western blot analysis and immunohistochemistry are powerful tools for evaluating GGT levels and activities in tissues, these laborious techniques are time-consuming and highly dependent on the quality of antibodies (Xu et al., 2018). Because of the simple preparation and manipulation, high spatiotemporal resolution and non-invasiveness method, fluorescent probes may offer an attractive alternative for conventional techniques (Yao et al., 2000; Zeng et al., 2007; Yang and Aghi, 2009; Yang et al., 2013; Zhao et al., 2015; Zhai et al., 2017). In this study, we have explored the possibility of using an *in situ* targeting probe as a biomarker for identification of various types of tissues based on GGT activity mapping. To the best of our knowledge, this is the first probe to be used for precise glioma pathology diagnosis through racking of GGT activity.

## Conclusion

Our results identified GGT as a potential biomarker for differentiating glioma recurrence and post-radio/chemotherapy mixtures for optimal malignancy evaluation. Importantly, it is more reliable to employ a GGT fluorescence probe for precise pathology evaluation. This procedure could easily be integrated into the current work-flow of neuro-navigation procedures to decrease the probability of missing biologically significant lesions in these high-risk glioma patients. Furthermore, we believe that this novel technique will facilitate research surrounding the intrinsic glioma progression mechanism with “precise” recurrence sample acquisition.

## Ethics Statement

All patients provided written informed consent. This study was approved by the Shandong University Institutional Review Board (NO.2015-051).

## Author Contributions

YL and CZ were guarantors of integrity of the entire study and responsible for manuscript editing. BS, ZZ, and CZ were responsible for the study concept and design. CL and YL were responsible for the experimental data analysis and statistical analysis. BW, SX, and MG were responsible for the clinical studies. GX and TZ were responsible for the literature research. BS, ZZ, and CL were responsible for the manuscript preparation.

## Conflict of Interest Statement

The authors declare that the research was conducted in the absence of any commercial or financial relationships that could be construed as a potential conflict of interest.
