# Supplementary material for: Enhanced γ-Glutamyltranspeptidase Imaging That Unravels the Glioma Recurrence in Post-radio/Chemotherapy Mixtures for Precise Pathology via Enzyme-Triggered Fluorescent Probe
Source: Front Neurosci. 2019 May 31;13:557. doi: 10.3389/fnins.2019.00557 (PMC6554337; doi:10.3389/fnins.2019.00557)
Supplement: Supplementary file 1 [file Data_Sheet_1.PDF]

Supplementary Material

1. Supplementary Files

1. 1 Study protocol.

Patients were examined by Gd-DTPA-enhanced MRI at a maximum of 1 week prior to surgery using automatic registration tools based on preoperative 3D T1 magnetization-prepared rapid acquisition gradient echo (3D-MPRAGE) scan. All patients then underwent neuro-navigation assisted tumor resection. First, a typical white light microscope resection was carried out under neuro-navigation guidance to completely remove the recurrent tumor as judged by the surgeon. After selection of tissue for pathology by the neurosurgeon, according to clinical experience, the rest of the specimen was transferred to liquid nitrogen for future imaging with the IVIS living system. Tissues that were identified using the GGT-responsive fluorescent probe were harvested and fixed for pathology diagnosis.

1.2 Cell viability assay.

NHA cells were incubated with the fluorescent probe CN-BOD-GSH (100 μM) for 12 and 24 h. Next, 10 μL of CCK-8 (Dojindo, Japan) solution was added to the mixture and incubated for another 4 h. The optical density at 450 nm was determined using a microplate reader.

1.3 Western blot analysis.

Cell lysates (100 μg protein from 10^6 U87R, U251R and NHA cells) or glioma samples (300 μg) were separated on a 10% SDS-PAGE gel and then transferred onto a nitrocellulose membrane. The membranes were then incubated in blocking buffer (5% skim milk) for 1 h, followed by treatment with primary antibodies overnight at 4˚C. Primary antibodies were GGT-1 (Sigma, SAB1405865, 1:100) and GAPDH (Santa Cruz, SAB1405865,as the endogenous control). The membranes were rinsed and incubated in horseradish peroxidase-conjugated secondary antibody for 1 h. Protein bands were visualized by using enhanced chemiluminescence (ECL) using western blot detection system (Bio-Rad Laboratories, Hercules, CA, USA).

2. Supplementary Figures

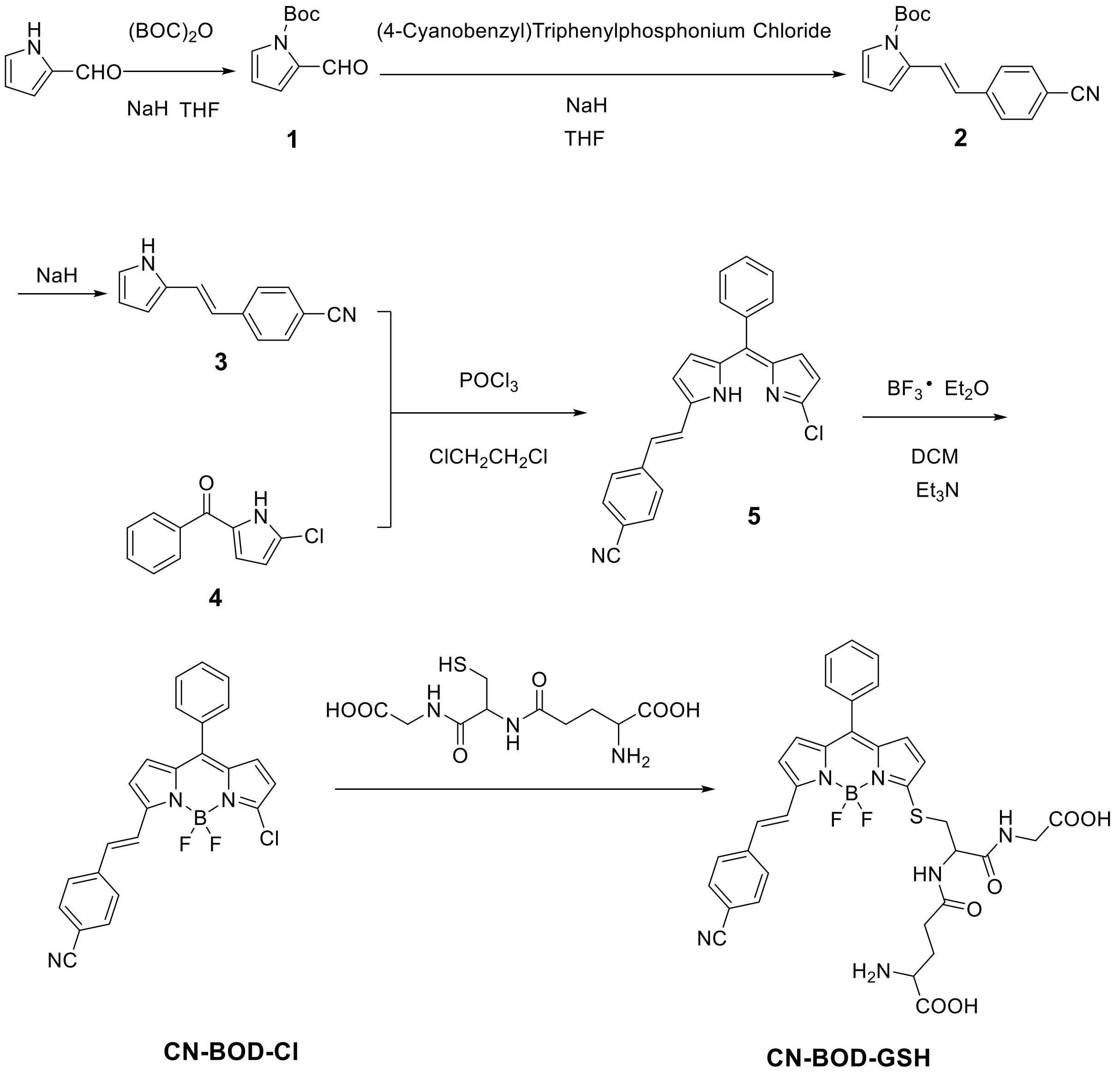

2.1 Supplementary Figure 1. Work-flow of the Synthesis of the CN-BOD-GSH probe.

Elemental Composition Report

Single Mass Analysis

Tolerance = 30.0 PPM / DBE: min = -1.5, max = 100.0
Element prediction: Off
Number of isotope peaks used for i-FIT = 2

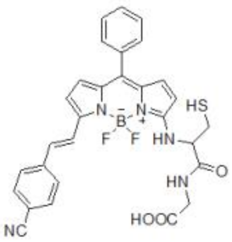

Monoisotopic Mass, Even Electron Ions
545 formula(e) evaluated with 1 results within limits (up to 1 best isotopic matches for each mass)
Elements Used:
C: 0-29 H: 0-24 N: 0-5 O: 0-3 S: 0-1 F: 0-2 K: 0-1 10B: 0-1

CC-ZHAO ECUST institute of Fine Chem 17-Jan-2018 21:46:57

ZC-ZZY-001 112 (1.464) Cm (111:112) 1: TOF MS ES+ 2.03e+000

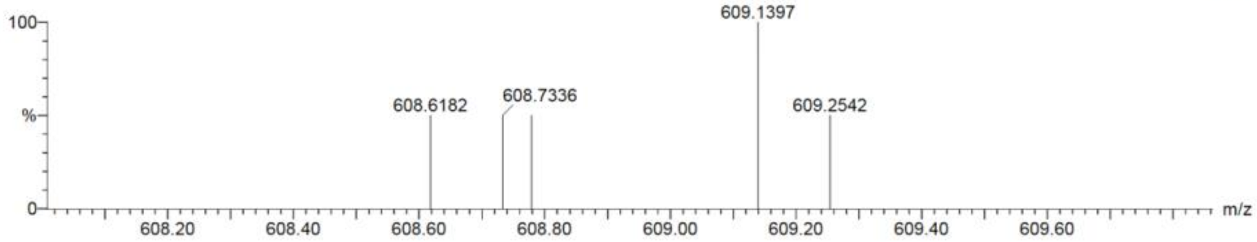

Minimum:				-1.5			
Maximum:		30.0	30.0	100.0			

Mass	Calc. Mass	mDa	PPM	DBE	i-FIT	i-FIT (Norm)	Formula
609.1397	609.1334	6.3	10.3	19.5	12.1	0.0	C29 H24 N5 O3 S F2 K 10B

2.2 Supplementary Figure 2. HRMS for confirmation of GGT catalyzed transformation of CN-BOD-GSH to CN-BOD-N ([M +K]$^+$ = 609.1397). HRMS was obtained at 40 min post addition of GGT to the solution of CN-BOD-GSH.

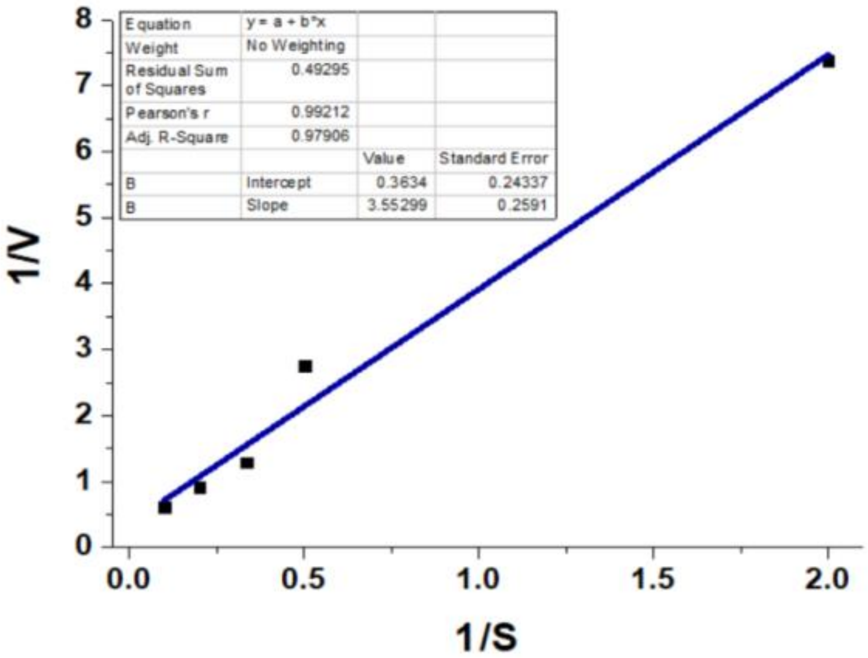

2.3 Supplementary Figure 3. Linear correlation between 1/V and of 1/S. Equation (1) and (2) can be derived from the Michaelis-Menten equation, where V represents the velocity, S is the substrate concentration and K_m is the Michaelis constant. From the linear plot, the slope and the intercept can be determined. K_m and V_{max} were calculated to be 9.78 μM and 2.75 μM • min^{-1}, respectively.

$$V = V_{max} \times [S]/(K_m + [S]) \quad (1)$$

$$1/V = (K_m/V_{max}) \times (1/[S]) + 1/V_{max} \quad (2)$$

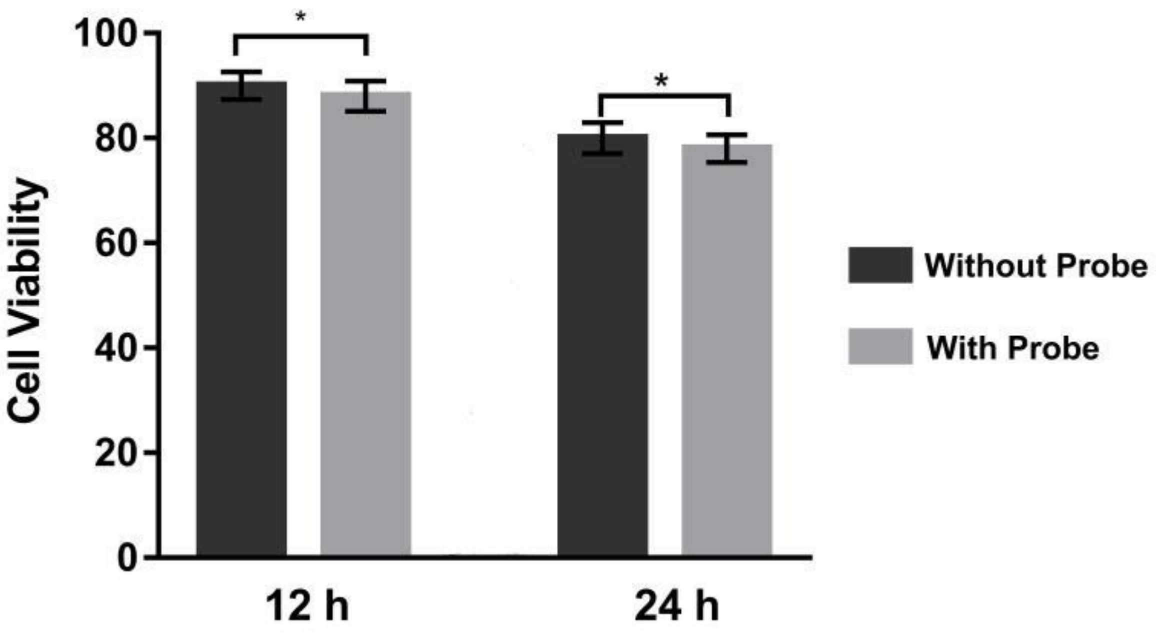

2.4 Supplementary Figure 4. CCK-8 cell viability assay in NHA cells treated with 100 μM of the CN-BOD-GSH probe for 12 h and 24 h. Experiments were performed in triplicate; Statistics analysis are performed by Student's t -test with the significance of P-valuation, *P>0.05.

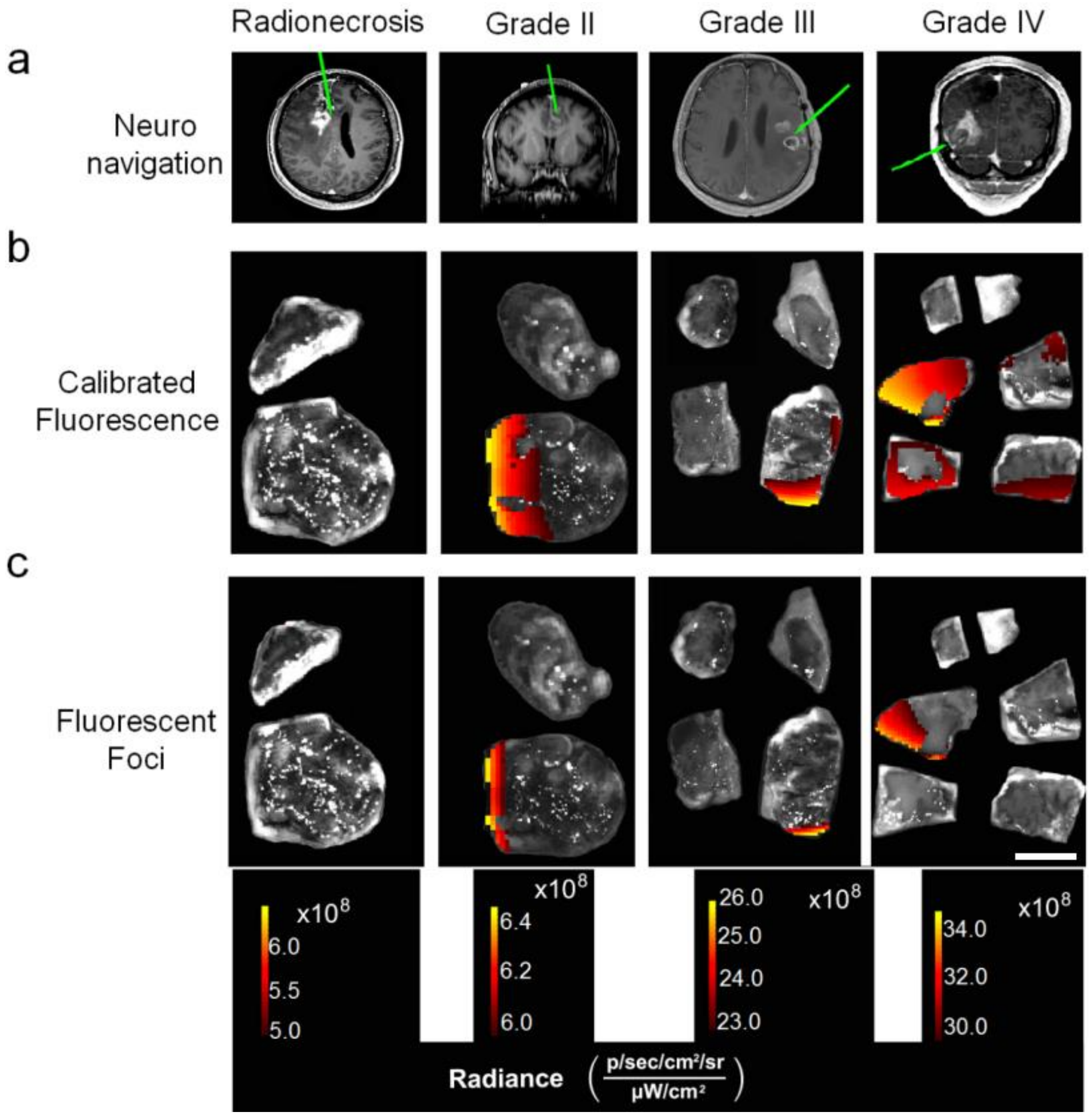

2.5 Supplementary Figure 5. (a) Tissues resected under neuro-navigation guidance after post-radio/chemotherapy. (b) The calibrated fluorescence in tissues after subtraction of the background fluorescence of the adjacent healthy tissue. (c) Continuous subtraction of background fluorescence in experimental tissues identifies the fluorescent foci for pathological diagnosis. Scale bars 10mm for all fluorescence images.

Supplementary Tables

Table S1. Patient's Information, pathological diagnosis and prognosis.

Patient ID	Sex	Age	Traditional diagnosis	WHO Grade	Ki-67 (%)	Fluorescent Foci	Diagnosis with CN-BOD-GSH	WHO Grade	Ki-67 (%)	Survival (Months)
T01	M	54	radionecrosis	0	4	No	radionecrosis	0	4.5	8
T02	F	62	radionecrosis	0	3	No	radionecrosis	0	2	10
T03	F	33	radionecrosis	0	4.5	No	radionecrosis	0	3.4	16
T04	F	35	AC	2	12	Yes	AC	2	9.1	26
T05	M	16	AC	2	6	Yes	AC	2	7.4	16
T06	F	49	AOA	3	10	Yes	AOA	3	13	20
T07	F	48	AOA	3	16	Yes	AOA	3	21	9
T08	M	62	AA	3	14	Yes	GBM	4	12	10
T09	F	56	AA	3	12	Yes	AA	3	18.5	6
T10	F	49	Post-irradiation	0	5	Yes	AOA	3	15	10
T11	F	57	GBM	4	18	Yes	GBM	4	20	6
T12	M	41	AA	3	10	Yes	GBM	4	15	7
T13	F	42	GBM	4	17	Yes	GBM	4	22	6
T14	M	65	GBM	4	22	Yes	GBM	4	18	8
T15	F	43	GBM	4	20	Yes	GBM	4	25	5
T16	F	57	GBM	4	15	Yes	GBM	4	30	5
T17	M	35	AC	2	8	Yes	GBM	4	42	8
T18	F	43	GBM	4	12	Yes	GBM	4	15	5
T19	F	49	GBM	4	19	Yes	GBM	4	35	8
T20	M	13	GBM	4	15	Yes	GBM	4	30	7

AC: astrocytoma; AA: anaplastic astrocytoma; AOA: anaplastic oligodentroastrocytoma; GBM: glioblastoma multiforme.
